# Novel Amphiphilic, Biodegradable, Biocompatible, Thermo-Responsive ABA Triblock Copolymers Based on PCL and PEG Analogues via a Combination of ROP and RAFT: Synthesis, Characterization, and Sustained Drug Release from Self-Assembled Micelles

**DOI:** 10.3390/polym10020214

**Published:** 2018-02-22

**Authors:** Wenyan Ning, Pei Shang, Jie Wu, Xiaoyu Shi, Shouxin Liu

**Affiliations:** Key Laboratory of Applied Surface and Colloid Chemistry, Ministry of Education, School of Chemistry and Chemical Engineering, Shaanxi Normal University, Xi’an 710062, China; 18792866293@163.com (W.N.); sp13772442791@163.com (P.S.); 18375847342@163.com (J.W.); 18647847370@163.com (X.S.)

**Keywords:** amphiphilic, ABA triblock copolymer, thermo-responsive, self-assembly, micellar drug release, sol-gel transition

## Abstract

Well-defined novel, linear, biodegradable, amphiphilic thermo-responsive ABA-type triblock copolymers, poly[2-(2-methoxyethoxy) ethyl methacrylate-*co*-oligo(ethylene glycol) methacrylate]-*b*-poly(*ε*-caprolactone)-*b*-poly[2-(2-methoxyethoxy) ethyl methacrylate-*co*-oligo(ethylene glycol) methacrylate] [P(MEO_2_MA-*co*-OEGMA)-*b*-PCL-*b*-P(MEO_2_MA-*co*-OEGMA)] (tBPs), were synthesized via a combination of ring-opening polymerization (ROP) of *ε*-caprolactone (*ε*CL) and reversible addition-fragmentation chain transfer polymerization (RAFT) of MEO_2_MA and OEGMA comonomers. The chemical structures and compositions of these copolymers were characterized using Fourier transform infrared spectroscopy (FT-IR) and proton nuclear magnetic resonance (^1^H NMR). The molecular weights of the copolymers were obtained using gel permeation chromatography (GPC) measurements. Thermo-responsive micelles were obtained by self-assembly of copolymers in aqueous medium. The temperature sensitivity and micelllization behavior of amphiphilic triblock copolymers solutions were studied by transmittance, fluorescence probe, surface tension, dynamic light scattering (DLS) and transmission electron microscopy (TEM). A hydrophobic drug, anethole, was encapsulated in micelles by using the dialysis method. The average particle sizes of drug-loaded micelles were determined by dynamic light scattering measurement. In vitro, the sustained release of the anethole was performed in pH 7.4 phosphate-buffered saline (PBS) at different temperatures. Results showed that the triblock copolymer’s micelles were quite effective in the encapsulation and controlled release of anethole. The vial inversion test demonstrated that the triblock copolymers could trigger the sol-gel transition which also depended on the temperature, and its sol-gel transition temperature gradually decreased with increasing concentration. The hydrogel system could also be used as a carrier of hydrophobic drugs in medicine.

## 1. Introduction

The synthesis of amphiphilic block copolymers by reversible addition-fragmentation chain transfer (RAFT) polymerization is the most convenient method [1,2]. These block polymers contain two parts of hydrophilic and hydrophobic segments, in aqueous solution, there will be self-assembly into core-shell structure micelles by the hydrophobic part as the core, hydrophilic part as the shell. The polymer solution occurs phase transformation when is stimulated by external environment, such as temperature, pH, light etc., and the external hydrophilic chains become aggregated and contracted [3,4,5]. Thus, they have been showing a series of interesting properties of solution [6]. Potential applications of such copolymers include drug delivery [7], coating [8,9], and colloid stabilization [10,11]. According to the literature, the widely studied thermo-responsive hydrophilic polymers are poly(*N*-isopropylacrylamide) (PNIPAAm) [12,13], poly(*N,N*-diethylaminoethyl methacrylate) (PDEAM) [14], poly(ethylene oxide) (PEO) [15], poly(2,5-diphenyloxazole) (PPO) [16], poly(dimethylaminoethyl methacrylate) (PDMAEMA) [17], poly(2-(2-methoxy ethoxy) ethylmethacrylate) (PMEO_2_MA) and poly(oligo (ethylene glycol) methacrylate) (POEGMA) [18,19,20], and so on.

Poly(*ε*-caprolactone) (PCL) is an US Food and Drug Administration (FDA) approved hydrophobic polymer. It is biocompatible, biodegradable nontoxic and widely used in various biomedical applications [21,22]. Compared with poly (lactic acid) (PLA) and poly (glycolic acid) (PGA), PCL-based polymers have excellent properties due to its crystallinity and hydrophobicity [23], and its degradation products do not substantially lead into acidic environments [24]. PCL-based amphiphilic copolymers are advantageous for drug delivery systems and PCL serves as the hydrophobic core, which can be used to load water-insoluble hydrophobic drugs [25,26,27]. It also presents slow degradation and provides a sustained release of drugs [28]. However, hydrophobicity goes against the potential application in drug delivery to take advantage of the benefits offered by PCL. In this regard, researchers modified the PCL with a hydrophilic segment and improved the hydrophobicity of the polymer to obtain an amphiphilic block polymer containing a hydrophobic PCL block as a hydrophobic core and hydrophilic block as a hydrophilic shell which have been reported [29,30,31,32,33]. So far, thermo-responsive amphiphilic block copolymers based on poly (ε-caprolactone)/poly(*N*-isopropylacylamide) (PCL/PNIPAAm) copolymers are the most studied for biomedical applications [29,30,31]. These amphiphilic polymers are soluble in water and self-assemble into micelles which usually have small sizes with low critical micelle concentration (CMC) allowing them to avoid the reticuloendothelial system uptake thereby promoting prolonged blood circulation [34]. The outer shell chains assume a random coil structure (hydrophilic state) below the lower critical solution temperature (LCST). When the temperature is increased above the LCST, it dehydrated and a collapsed globular structure (hydrophobic state) formed, allowing aggregation of micelles [35]. Recently, the copolymers of 2-(2-methoxyethoxy) ethyl methacrylate (MEO_2_MA) and oligo(ethylene glycol) methacrylate (OEGMA) have been drawing a lot of attention as a new family of thermo-responsive polymers. These PEG analogues exhibited a LCST between 26 °C and 90 °C which can be precisely adjusted by varying the co-monomers ratio [18]. The delicate balance between hydrophilic and hydrophobic segments in polymer and the hydrogen bonding interaction between ether oxygen bond and solvent determine the LCST values of P(MEO_2_MA-*co*-OEGMA) [25]. The copolymer P(MEO_2_MA-*co*-OEGMA) could be attractive for controlled drug delivery because of their thermo-responsive properties, good hydrophilicity, and outstanding biocompatibility, nontoxicity and by FDA approved similar to linear PEG. PNIPAAm also has good temperature response, but it is short of biocompatibility. In addition, their LCST values were found to be less influenced by factors as ionic strength, concentration of the copolymer in water, and chain length, so that not be adjusted in a large range [20,30]. Therefore, the P(MEO_2_MA-*co*-OEGMA) based copolymers were used as alternative to PNIPAAm. Diblock and triblock copolymers containing MEO_2_MA and OEGMA have been synthesized previously [18,36], but not containing a central biodegradable, hydrophobic block of PCL. Later, the synthesis and micellization of PCL/P(MEO_2_MA-*co*-OEGMA) copolymers will been studied. In fact, no drug release data have been reported.

**Scheme 1:**
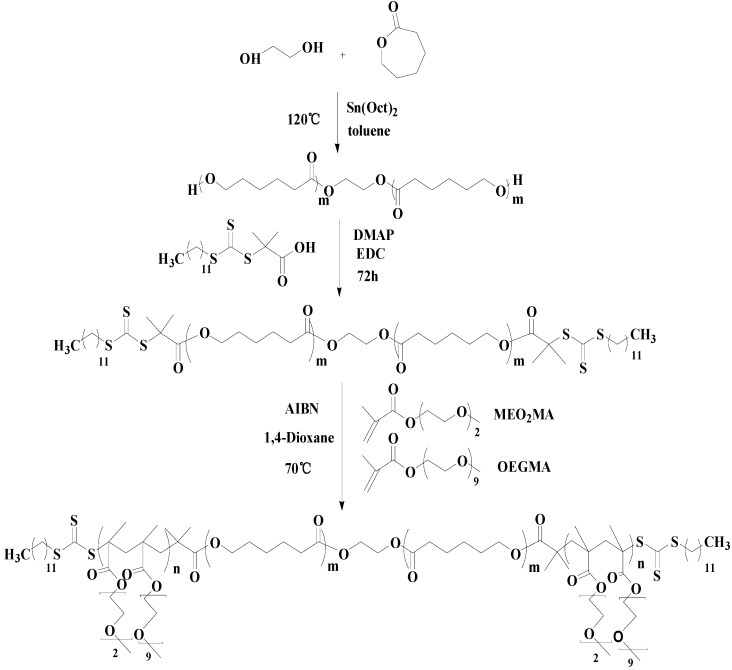
Synthesis of triblock copolymer P(MEO_2_MA-*co*-OEGMA)-*b*-PCL-*b*-P(MEO_2_MA-*co*-OEGMA).

In this work, a series of ABA-type amphiphilic triblock copolymers composed of a biodegradable PCL central block and two outer thermo-responsive P(MEO_2_MA-*co*-OEGMA) blocks were prepared by a combination of ring opening polymerization (ROP) and reversible addition-fragmentation chain transfer (RAFT) polymerization methods to solve the solubility of PCL (Scheme 1). The chain length of the central PCL block was maintained constant and varied that of the outer fragments of P(MEO_2_MA-*co*-OEGMA) for comparison. The well-defined amphiphilic P(MEO_2_MA-*co*-OEGMA)-*b*-PCL-*b*-P(MEO_2_MA-*co*-OEGMA) triblock copolymers were synthesized with a high yield. The micellization behavior, aqueous solution aggregation behavior of the obtained amphiphilic block copolymers were studied using UV transmittance, fluorescence spectroscopy, light scattering, and transmission electron microscopy. The potential of PCL/PEG analogues micelles as thermo-sensitive drug carrier and temperature-dependent sol-gel transition were also investigated. Anethole is a typical hydrophobic drug whose function is to increase neutrophils for leukocytopenia due to chemotherapy and radiotherapy, as well as leukocytopenia caused by other reasons. Anethole was chosen as a model drug that was encapsulated in the polymeric micelles by using dialysis method. For the first time, thermo-responsive drug release in vitro of the polymeric micelles was investigated at 25 and 37 °C.

## 2. Experimental Section

### 2.1. Materials

*ε*-Caprolactone (*ε*CL) and stannous octoate (Sn(Oct)_2_, 96%) were purchased from Alfa Aesar (Shanghai, China). ε-Caprolactone (εCL) was dried over calcium hydride (CaH_2_) for 48 h at room temperature and distilled under reduced pressure just before use. Ethylene glycol (Guo Yao Chemical Company, Shanghai, China, 99%) was dried over CaO and then distilled under a reduced pressure. *N*-(3-(dimethylamino) propyl)-*N*-ethylcarbodiimide hydrochloride (EDC) and 4-(*N*,*N*-dimethylamino)pyridine (DMAP) were purchased from TCI (Shanghai Development Co., Ltd., Shanghai, China). 2-(2-methoxyethoxy) ethyl methacrylate (MEO_2_MA, 95%, *M*_n_ = 188.22 g·mol^−1^), oligo(ethylene oxide)methacrylate (OEGMA, 95%, *M*_n_ = 475 g·mol^−1^) and trans-anethole were obtained from TCI (Shanghai Development Co., Ltd., Shanghai, China). *N*,*N*-azobisisobutyronitrile (AIBN) was recrystallized from methanol. All solvents were distilled prior to use. CH_2_Cl_2_ was dried over CaH_2_, Toluene and 1,4-dioxane was refluxed over sodium and distilled. Double-distilled water was used for preparing all aqueous solutions. All the other reagents were acquired from commercial sources and used without further purification.

### 2.2. Characterization

#### 2.2.1. Nuclear Magnetic Resonance Spectroscopy (^1^H NMR)

^1^H NMR spectra are recorded on A Bruker Avance 300 MHz upper-conducting Fourier Digital nuclear magnetic resonance instrument (^1^H NMR) (300 MHz Avance, Bruker Corporation, Karlsruhe, Germany) at room temperature in CDCl_3_ or D_2_O as the solvent. Chemical shifts (*δ*) relative to tetramethylsilane (TMS) were given.

#### 2.2.2. Gel Permeation Chromatography (GPC)

The number average molecular weight (*M*_n_) and polydispersity index (*M*_w_/*M*_n_) are determined by gel permeation chromatography (GPC) (Breeze, Waters, Milford, MA, USA) in THF at 25 °C with a flow rate of 1 mL/min, and monodisperse polystyrene was used as the standard for calibration. A series of polymer solutions in THF were prepared at a concentration of 4 mg/mL and were filtered through an oil phase pin-type filter of 0.45 μm diameter prior to measurement.

### 2.3. Synthesis

#### 2.3.1. Synthesis of Dihydroxy-Terminated Poly(ε-caprolactone) (HO-PCL-OH)

Synthesis of HO-PCL-OH used the method of ring opening polymerization (ROP) with ethylene glycol as the initiator and tin(II) 2-ethylhexanoate(Sn(oct)_2_) as the catalyst [29]. The synthetic route is shown in Scheme 1 (Step 1) and the specific synthesis process was as follows. Ethanediol (62 mg, 1 mmol) and εCL (3.42 g, 30 mmol) (initiator/monomer ratio 1:30) were taken in a dry three-necked flask equipped with a magnetic stirring bar under a nitrogen atmosphere. After, Sn(Oct)_2_ (20 μL, 0.05 mmol) and dry toluene solvent were added to the above mixture, respectively. Then, the flask was heated on an oil bath at 120 °C with vigorous stirring for 24 h under protection of the nitrogen atmosphere. After the reaction was finished, the remaining solvent was removed by rotary evaporation. After concentration, the solution was precipitated in excess of cold hexane for two times, then dried in a vacuum oven overnight at room temperature, yielding white solids.

#### 2.3.2. Synthesis of the Macro-RAFT Agent (CTA-PCL-CTA)

The synthesis of *S*-1-dodecyl-*S*′-(α,α′-dimethyl-α″-acetic acid) trithiocarbonate chain transfer agent (CTA) was carried out according to the method of the literature [37]. Synthesis of CTA-PCL-CTA used esterification, the synthetic route of CTA-PCL-CTA is shown in Scheme 1 (Step 2). *S*-1-dodecyl-*S*′-(α,α′-dimethyl-α″-acetic acid) trithiocarbonate chain transfer agent CTA (3.276 g, 9 mmol), HO-PCL_30_-OH (3.486, 1 mmol), and catalytic amount of DMAP (0.055 g, 0.45 mmol) were dissolved in 40 mL methylene dichloride. When the solution was homogenized by stirring, EDC (1.725 g, 9 mmol) was added in one portion into the solution. The esterification reaction proceeded under stirring at room temperature for 48 h. The by-product was washed in Na_2_CO_3_ aqueous solution (3 × 100 mL). The organic layer is further washed with water (2 × 100 mL), Then the organic layer solution was dried 24 h in anhydrous sodium sulfate, the solution was filtered and evaporated on the rotary evaporator, and the concentrated liquid was dripped into the cold ether to precipitate, and finally the product was obtained by vacuum drying [37,38]. Macro-RAFT agent (CTA-PCL-CTA) with yellow color was obtained by filtration and dried under vacuum at room temperature for 24 h.

#### 2.3.3. Synthesis of P(MEO_2_MA-*co*-OEGMA)]-*b*-PCL-*b*-P(MEO_2_MA-*co*-OEGMA) Copolymers 

The triblock copolymer P(MEO_2_MA-*co*-OEGMA)]-*b*-PCL-*b*-P(MEO_2_MA-*co*-OEGMA) was prepared by reversible addition fragmentation chain transfer polymerization (RAFT). The synthetic route is shown in Scheme 1 (Step 3) and in a typical experiment, firstly, macroinitiator CTA-PCL-CTA (0.4178 g, 0.1 mmol), AIBN (0.0082 g, 0.05 mmol) were added to 5 mL 1,4-dioxane in a Schlenk flask and stirred to form a homogeneous solution under a nitrogen atmosphere. Next, a mixed solution containing MEO_2_MA (3.94 mL, 21.34 mmol) and OEGMA (0.29 mL, 0.66 mmol) was added into the reaction mixture in order to prepare the P(MEO_2_MA-*co*-OEGMA) block. The reaction tube was sealed and placed in an oil bath at 70 °C with vigorous stirring for 12 h under the protection of nitrogen atmosphere. The polymerization was stopped by exposing the reaction system to air. The final mixture was diluted by methanol, and the solution was dialyzed against demonized water for four days using a MWCO 8–14 kDa dialysis bag to remove catalyst, any small molecules, the monomer and oligomer. After freeze drying, the triblock copolymer was obtained.

### 2.4. Transmittance Measurements

The transmittance of the triblock copolymers aqueous solution was monitored at a wavelength of 500 nm with a UV-VIS spectroscopy (TU-1901, Beijing Purkinje General Instrument Corporation, Beijing, China) with a Peltier temperature-controlled system. The temperature ramp was 1 °C·min^−1^. The cloud point was identified from 50% of the transmittance versus the temperature plot.

### 2.5. Critical Micellization Concentration Determination (CMC)

The CMC of the copolymers was determined by fluorescence spectroscopy using pyrene as a hydrophobic fluorescent probe. Measurements were carried out on a PELS55 fluorescence spectrophotometer (PELS55, America Perkin Elmer Corporation, Waltham, MA, USA). The triblock copolymer concentrations varied from 1.0 × 10^−3^ to 0.1 mg·mL^−1^. Measurement conditions were set as follows: the excitation wavelength was at 331 nm and the emission spectra recorded range was from 350 to 500 nm. The ratios of the peak intensities at 384 and 373 nm (*I*_384_/*I*_373_) of the emission spectra were recorded and plotted the *I*_384_/*I*_373_ ratio versus polymer logarithmic concentration, the CMC value was determined from the intersection of linear regression lines on plots. In addition, the surface tension method could be used to determine the CMC value of the polymer solution using an automatic surface tension meter (DCAT 21, Germany Dataphysics Corporation, Stuttgart, Germany). Plotting surface tension versus polymer concentration and obtaining the intersection point by the extrapolation resulted in the CMC of the polymer solution.

### 2.6. Dynamic Light Scattering (DLS)

The particle size and particle size distribution of the triblock block polymer solutions were measured by dynamic light scattering (DLS) on a Malvern Zetasizernano ZS90 (Otsuka electronics, Osaka, Japan) with a 4 mW He-Ne solid state laser. The laser wavelength was 633 nm, the scattering angle was 90°, and the temperature range was 25–50 °C. All polymer solutions of 2 mg·mL^−1^ were prepared and impurities were removed with a water system filter with an aperture of 0.45 μm microns.

### 2.7. Transmission Electron Microscopy (TEM)

TEM experiments were carried out on a JEOL JEM-2100 instrument (Tokyo, Japan) with an accelerating voltage 200 kV. The samples were prepared by dropping a polymer solution at 2.0 mg·mL^−1^ at different temperatures onto a carbon coated copper grid (300 mesh), stained with 1% (*w*/*v*) phosphotungstic acid (PTA) solution, and followed by drying in the corresponding temperatures.

### 2.8. Sol-Gel Transition

The formation of gel and critical temperature in gel-to-sol transition (CGT) were measured via the test tube inverting method. The triblock copolymers were added into a vial (5 mL) and were dissolved at a given concentration in distilled water stirring for three days at room temperature. Then the vial was immersed in a water bath and allowed to equilibrate at every temperature from 25 to 50 °C for 15 min. Then the small bottles were tilted or inverted to observe their physical states. The sol-gel transition temperatures of the samples were determined by the inversion of the small bottles by 180° for 1 min, and if the solution did not flow, it was recognized as a stable gel. The temperature was recorded as the sol-to-gel transition temperature.

### 2.9. Preparation of Anethole-Loaded Polymeric Micelles

The anethole-loaded micelle was obtained by combination of solvent evaporation and dialysis methods [27]. Firstly, 2 mg of the anethole and 20 mg of the dry copolymer of P(MEO_2_MA-*co*-OEGMA)]-*b*-PCL-*b*-P(MEO_2_MA-*co*-OEGMA)(tBP3) was dissolved completely in 3 mL DMF in a glass vial at room temperature. Afterwards, the solution was added dropwise to 20 mL deionized water under vigorous stirring, yielding self-assembled micelles at room temperature. The crude anethole-loaded micelle solution was centrifuged (TGL-16M, in China) for 10 min at a speed of 7980 rpm to remove the undissolved anethole and aggregated micelles, and then the supernatant was further purified by dialysis against water for 48 h using a MWCO 8–14 kDa dialysis bag to eliminate free anethole and DMF. The fresh water was replaced every 4 h. The anethole-loaded micelle solution was lyophilized under vacuum for three days and then stored at 4 °C before use.

To determine the drug loading (DL) and encapsulation efficiency (EE), a known amount of freeze-dried anethole-loaded micelles was dissolved in DMF and the absorbance was measured on a UV-VIS spectrophotometer (TU-1901, Beijing, China) at 258 nm. The calibration curve is obtained by using different concentrations of free anethole in DMF. The amounts of anethole loaded and encapsulated in micelles were calculated according to the standard curve of the anethole-DMF solution. The DL and EE were calculated from following formulae:

$$\mathrm{DL}\;(\%)=\frac{\mathrm{weight}\;\mathrm{of}\;\mathrm{loaded}\;\mathrm{drug}}{\mathrm{weight}\;\mathrm{of}\;\mathrm{polymer}}\times 100$$

$$\mathrm{EE}\;(\%)=\frac{\mathrm{weight}\;\mathrm{of}\;\mathrm{loaded}\;\mathrm{drug}}{\mathrm{theoretical}\;\mathrm{drug}\;\mathrm{loading}}\times 100$$

### 2.10. In Vitro Drug Release

The release of drug-loaded micelles in vitro was studied by the dialysis method and evaluated in 200 mL of phosphate-buffered saline (PBS, 0.2 M, pH = 7.4) against 5 mL of anethole-loaded micelle (1 mg·mL^−1^) solution that was loaded in a dialysis bag (MWCO 2000) beforehand, and the dialysis bag was shaken (90 rpm) at 25 and 37 °C. At predetermined time intervals, 3.0 mL the release medium was withdrawn and replaced immediately by the same amount of fresh buffer medium. The anethole concentration was determined by using a UV-VIS spectrophotometer (TU-1901, Beijing, China) based on the absorbance intensity at 258 nm against a calibration curve. The calibration curve used for drug release was obtained by using different concentrations (1–50 mg·mL^−1^) of free anethole in PBS. The accumulative release (%) was calculated following the formula:

$$\mathrm{Cumulative}\;\mathrm{release}\;(\%)=\frac{V_{e}\;\sum_{1}^{n-1}c_{i}+V_{0}c_{n}}{m_{\mathrm{drug}}}\times 100$$

where *V*_e_ and *c*_i_ are the volume and concentration of the sample withdrawn at the interval of *t*_i_, *V*_0_ is the total volume of the release medium, *c*_n_ is the concentration of the sample withdrawn at the interval of *t*_n_, and *m*_drug_ is the total amount of anethole in the micelle. Three replicates were measured for each sample, and the results presented are the average data.

## 3. Results and Discussion

### 3.1. Synthesis and Characterization of the Polymers

The triblock copolymers P(MEO_2_MA-*co*-OEGMA)-*b*-PCL-*b*-P(MEO_2_MA-*co*-OEGMA) were synthesized via the combination of ROP and RAFT polymerization, as shown in Scheme 1. At first, HO-PCL-OH is synthesized via ROP of ε-CL in bulk at 120 °C using ethylene glycol as the initiator and Sn(Oct)_2_ as the catalyst. PCL is a highly-crystallized polymer and its crystallinity increases with increasing molecular weight. This fact slows the drug release [22]. Moreover, the molecular weight of PCL of ca. 2000 g·mol^−1^ was good enough for self-assembled micelles [39]. Therefore, we selected the DP of PCL ca. 30 in this work. Then, HO-PCL-OH is reacted with *S*-1-dodecyl-*S*′-(α,α′-dimethyl-α″-acetic acid) trithiocarbonate (CTA) in CH_2_Cl_2_ to prepare the CTA-*b*-PCL-*b*-CTA macro-chain transfer agents. The CTA-*b*-PCL-*b*-CTA is further used as a macro-chain transfer agent for the RAFT polymerization of MEO_2_MA, OEGMA in 1,4-dioxane using CTA-*b*-PCL-*b*-CTA/[AIBN] = 1:0.5 at 70 °C for 12 h (Scheme 1). The P(MEO_2_MA-*co*-OEGMA)-*b*-PCL -*b*-P(MEO_2_MA-*co*-OEGMA) triblock copolymers are prepared using different molar ratios of MEO_2_MA,OEGMA monomers (Table S1).

The chemical structures of the CTA, HO-*b*-PCL-*b*-OH, and macro-RAFT agent CTA-*b*-PCL-*b*-CTA were proven by ^1^H NMR and FT-IR spectra. Figure S1 shows the FT-IR spectra of the CTA, OH-*b*-PCL-*b*-OH, and CTA-*b*-PCL-*b*-CTA. In the FT-IR spectrum of the CTA (Figure S1a), the peaks at 3423, 1700 cm^−1^ (−OH and C=O stretching vibration) and 1065 cm^−1^ (C=S vibration in the trithiocarbonate units) demonstrate the existence of CTA. The FT-IR spectrum of the HO-PCL-OH is shown in Figure S1b, the peaks at 3680, 2945, 1471 cm^−1^ (−OH, −CH of CH_2_ stretching vibrations, and −CH in-plane bending vibrations) and 1731 cm^−1^ (C=O stretching vibration) show the existence of PCL. Compared with Figure S1a,b, the peak of hydroxyl group disappeared in the Figure S1c because the esterification reaction proceeds between the carboxyl of CTA and the hydroxyl of the PCL.

Figure S2Ab,Ac show the ^1^H NMR spectra of HO-PCL-OH and the as-prepared CTA-PCL-CTA macro-RAFT agent. In Figure S2Ab, the peak at 4.2 ppm (peak “a”) corresponding to the proton of −OC***H_2_***C***H_2_***OC=O, the peak at 2.3 ppm (peak “b”) corresponding to methylene groups of *ε*-CL unit (−OCH_2_CH_2_CH_2_CH_2_C***H_2_***C=O), the signal at 1.6 ppm (peak “c”) is assigned to the proton in the methylene of *ε*-CL unit (−OCH_2_C***H_2_***CH_2_C***H_2_***CH_2_C=O) and the peak at 1.4 ppm (peak “d”), the peak at 4.05 ppm(peak “e”) and the peak at 3.6 ppm (peak “f”) are attributable to the proton signals of methylene groups of *ε*-CL unit (−OCH_2_CH_2_C***H_2_***CH_2_CH_2_C=O, O=CCH_2_CH_2_CH_2_CH_2_C***H_2_***OC=O, and −OC***H_2_***CH_2_CH_2_CH_2_CH_2_C=O), respectively. In Figure S2Ac some new peaks are apparent in the ^1^H NMR spectrum, compared with that of HO-PCL-OH: the peak at 3.27 ppm (peak “h”), which corresponds to the proton of –C***H_2_***CH_2_–, and the signal at 1.73 ppm (peak “i + g”) which is assigned to the proton of –C(C***H_3_***)_2_– and the signal at 1.27 ppm and 0.90 ppm (peaks “j” and “k”) which was assigned to the proton in the methylene and terminal methyl of the CTA chain.

Figure S2Ba, typical ^1^H NMR spectrum of the triblock copolymer P(MEO_2_MA-*co*-OEGMA) -*b*-PCL-*b*-P(MEO_2_MA-*co*-OEGMA) in CDCl_3_ show that in addition to the characteristic peaks of the PCL block, the characteristic peaks at 4.0 ppm is assigned to the methylene protons (peak “*r*”) adjacent to the ester bond of both MEO_2_MA and OEGMA. Other methylene protons (peak “*p*”) of MEO_2_MA and OEGMA are observed at 3.6 ppm. The signal at 3.4 ppm is assigned to the methyl end group of MEO_2_MA and OEGMA [19]. Therefore, the thermosensitive triblock copolymer was synthesized. In addition, the molecular weight and molecular weight distribution of the polymers are determined by GPC. Characteristics of all the samples are summarized in Table S1.

On the other hand, the peaks of PCL block are suppressed in D_2_O (Figure S2Bb). Hence, CDCl_3_ is a good solvent for both PCL and P(MEO2MA-*co*-OEGMA), while water is a poor solvent for PCL. This observation indicates that the micelle aggregates are possibly formed in aqueous solution with PCL blocks as the core and P(MEO_2_MA-*co*-OEGMA) blocks as the shell.

### 3.2. Water-Solubility and Temperature Sensitivity of the Triblock Copolymers

**Figure 1:**
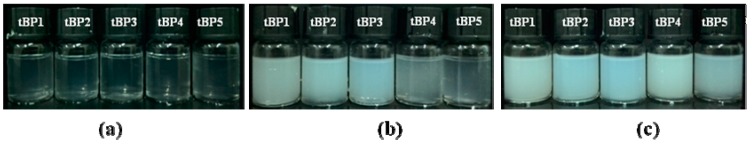
Photographs of the triblock copolymers aqueous solutions (2 mg·mL^−1^) at 25 °C (**a**), 35 °C (**b**), and 45 °C (**c**).

It was all know that the PCL is a kind of hydrophobic, biodegradable and semi-crystalline polymer, but its hydrophobicity limits its wider application. Owing to P(MEO_2_MA-*co*-OEGMA) is a hydrophilic thermo-responsive copolymer, while the PCL block is modified by P(MEO_2_MA-*co*-OEGMA) chains, the solubility of the PCL in water may be improved and might endow the P(MEO_2_MA-*co*-OEGMA)-*b*-PCL-*b*-P(MEO_2_MA-*co*-OEGMA) with amphiphilicity and temperature sensitivity. Figure 1 displays the photographs of the triblock copolymers’ aqueous solutions at different temperatures. In Figure 1a all aqueous solutions of the triblock copolymers were transparent at room temperature (25 °C) because the hydrophilic chain P(MEO_2_MA-*co*-OEGMA) is stretchable in the amphiphilic polymer. In addition, photographs of the tBPs aqueous solutions (tBP1, tBP2, tBP3) shows that with the increase of the degree of polymerization, aqueous solutions of polymers were brighter.

Then, at 35 °C in Figure 1b, the solutions were translucent. At this temperature, the P(MEO_2_MA-*co*-OEGMA) chains on PCL block collapse and aggregate since the hydrogen bond interactions of the copolymer with the surrounding water molecules began to break down, leading to a portion of the micelles to transform into aggregate nanoparticles. When the temperature continually increased in Figure 1c, the solutions turned into a white milky dispersion, owing to the hydrogen bonding between intermolecular segments being completely destroyed, and the hydrophobic effect was strong enough to induce a long-range interconnection of nanoparticles. In addition, observing solutions changes of tBP2, tBP4, and tBP5, the degree of change with the temperature is not the same due to the different content of OEGMA. All the results demonstrated that the triblock copolymers have superior solubility and temperature sensitivity. Moreover, water solubility for a triblock copolymer depends on the degree of polymerization of the P(MEO_2_MA-*co*-OEGMA) chains on PCL block and the content of OEGMA affects the phase transition temperature of the solutions.

**Figure 2:**
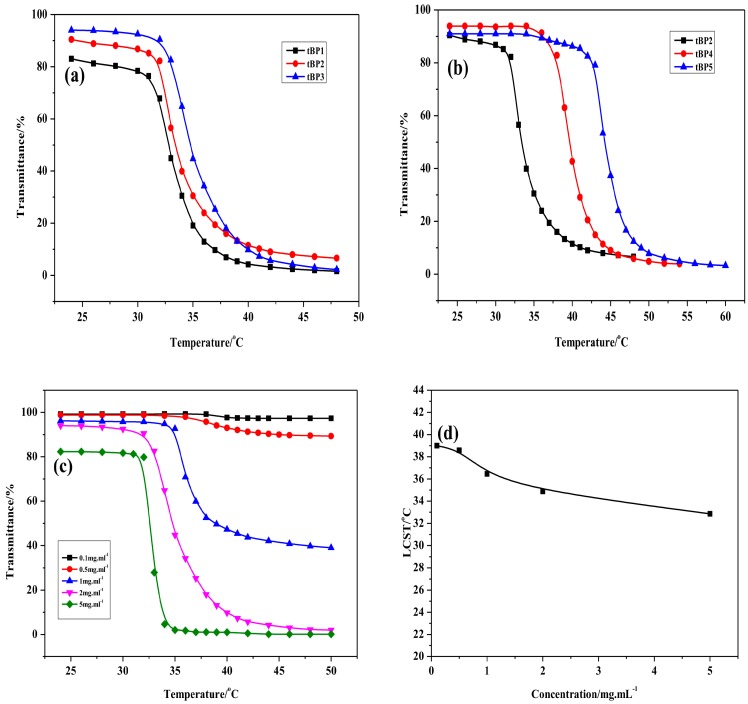
Curves of the triblock copolymers aqueous solutions transmittance versus temperatures with (**a**) different degrees of polymerization, (**b**) the different content of OEGMA, (**c**) different concentrations of tBP3 aqueous solutions, and (**d**) LCST versus the tBP3 concentration.

Particularly, temperature sensitivity for tBPs aqueous solutions could be verified via the change of solution transmittance versus temperature. Figure 2a, curves of the triblock copolymers aqueous solutions transmittance versus temperatures with different degrees of polymerization. It can be easily observed that the transmittance of the solutions gradually increases with the rise in degrees of polymerization of the P(MEO_2_MA-*co*-OEGMA) chains on PCL block from 220, to 250, to 280. Additionally, the LCSTs of tBP1, tBP2, and tBP3 are 33, 34 and 35 °C, respectively. It could be draw a conclusion that the LCST of the triblock copolymers rose with the increase in the degrees of polymerization of the P(MEO_2_MA-*co*-OEGMA) chains. Due to the introduction of the hydrophilic P(MEO_2_MA-*co*-OEGMA) block, the hydrogen bonds have formed between the side chains of oxethyl units and water molecules, resulting in an increase in the hydrophilicity of the polymer. The longer the hydrophilic chain is, the more energy is required to destroy the balance between the hydrophilic and hydrophobic interaction of the polymer. In other words, it is necessary to have a higher temperature, that is, the higher LCST.

Figure 2b shows the curves of the triblock copolymers aqueous solutions transmittance versus temperatures with different contents of OEGMA. The tBPs aqueous solutions (tBP2, tBP4, tBP5) shows that the LCST of the triblock copolymer aqueous solutions increases from 34 to 45 °C with the content of OEGMA increasing from 3% to 13%. Figure 1b,c, photographs for the tBPs (tBP2, tBP4, tBP5) aqueous solutions at 35 and 45 °C can also demonstrate that the contents of OEGMA affects the LCST of the triblock copolymers.

Figure 2c displays the temperature dependence of the transmittance of tBP3 aqueous solution at different concentrations ranged from 0.1 to 5 mg·mL^−1^. The results demonstrate that the LCST values of those aqueous solutions increase with decreasing concentrations. At concentrations of 0.1 and 0.5 mg·mL^−1^, the transmittance of the tBP3 aqueous solution has a small variation as a function of the temperature. For the concentration was greater than 2 mg·mL^−1^, the transmittance of the tBP3 aqueous solution was greatly reduced at room temperature due to self-aggregation. The results suggested that a suitable concentration that provided a sufficient amount of micelle aggregate particles without kinetically restricted aggregation during the thermally-induced phase transition was necessary for precise LCST measurement [25,26]. In addition, in the studied range of concentration (0.1–5 mg·mL^−1^), the LCST was found to only increase a few degrees with the dilution solution concentration (see Figure 2d). The LCST values of the tBP3 aqueous solution increased from 33 to 39 °C and shifted the cloud point 6 °C toward higher temperature with changing concentrations from 5 mg·mL^−1^ to 0.1 mg·mL^−1^. A similar phenomenon had been observed in other thermo-sensitive copolymers [18].

### 3.3. Micellization of the Triblock Copolymers 

**Figure 3:**
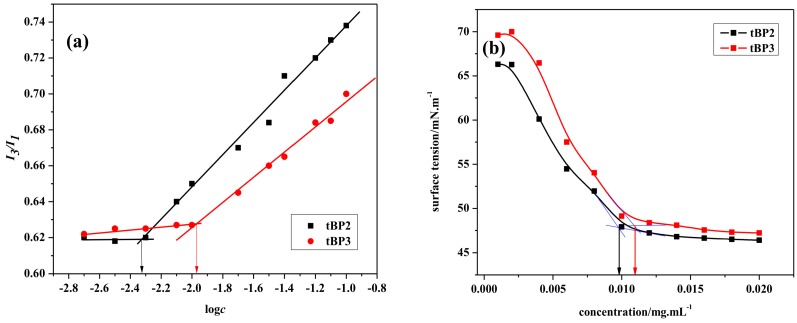
The CMC of the triblock copolymer tBP2 and tBP3 as determined by the emission spectra (**a**) or automatic surface tension meter (**b**).

The amphiphilic block copolymers in aqueous solution will self-assemble into micellar aggregates. The CMC is the lowest concentration of forming micelles which is an important parameter to investigate the properties of amphiphilic polymers and evaluate the stability of micelles as drug delivery carriers. In order to study the critical micellar concentration of such triblock copolymers in water, fluorescence spectroscopy is used with pyrene as the probe, or an automatic surface tension meter. Figure 3a shows the *I*_384_/*I*_373_ (*I*_3_/*I*_1_) ratio changes as a function of copolymer concentration. The intensity ratio exhibits a substantial increase at a particular concentration, indicating the incorporation of pyrene into the hydrophobic cores of the micelles. The CMC was obtained from the intersection of the two linear regression lines of the plots. The observed CMCs of the triblock copolymers tBP2 and tBP3 are about 0.0046 and 0.0105 mg·mL^−1^, respectively. The CMC is three times lower than that of P(MEO_2_MA-*co*-OEGMA)-PLLA-P(MEO_2_MA-*co*-OEGMA) copolymers micelles (0.0113–0.0130 mg·mL^−1^) reported by Hu, and five times lower than that of POEGMA-*b*-PCL-*b*-POEGMA micelles (0.0254–0.0372 mg·mL^−1^) reported by Luzon [19,40]. The CMC was close to that of PNIPAAm-*b*-PCL-*b*-PNIPAAm micelles (0.0015–0.0050 mg·mL^−1^) [29]. The very low CMC demonstrates the strong tendency of copolymers toward the formation of micelles, which is of major importance for the stability of micelles in long circulation in the bloodstream after injection induced dilution and is desired to avoid the dissociation of micelles during the dilution of drug delivery systems by body fluid [19].

In this work, by comparison, the CMC value of triblock copolymers increases with the increase in the chain length of the P(MEO_2_MA-*co*-OEGMA) block from 220 to 280, in addition to the value of CMC is related to the internal interaction of the polymer chains, accounting for the proportion of the hydrophilic and hydrophobic blocks of the polymer and external temperature [29]. For the amphiphilic block polymer, the longer the hydrophilic chain, the higher the CMC value will be, which is due to the solubility of the block copolymer in water the increase of the length of the hydrophilic chain. On the contrary, the longer the hydrophobic chain, the smaller the CMC value will be, because of the longer the hydrophobic chain, the stronger hydrophobicity will be, and the polymer solution is more easily self-assembled into forming micelles. Thus, the observed results are in conformity with this explanation. Similar type of results is also reported in the literature [29,31].

The automatic surface tension method is used to determine the CMC value of the polymer solution. The test results of CMCs are shown in Figure 3b. The acquisition of CMC is the intersection point of two linear regimes obtained through extrapolation. When the concentration is higher than its CMC, the amphiphilic triblock copolymer chains tend to aggregate to form micelles rather than migrate to the interface. As a result, the interfacial tension maintains an almost constant value above the CMC. The CMC value of the tBP3 is larger than that of the tBP2, which mainly corresponds to the CMCs measured by the fluorescence probe technique.

### 3.4. Size and Morphology of the Polymeric Micelles

**Figure 4:**
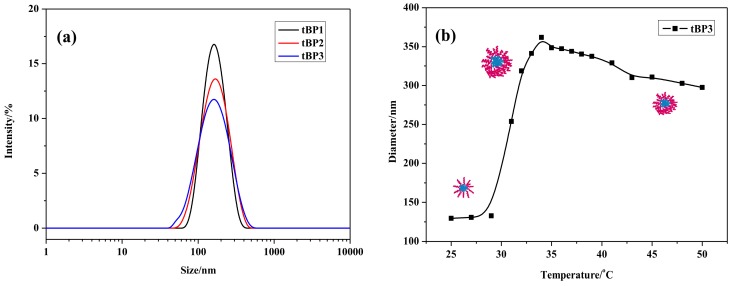
Size distributions of tBPs aqueous solutions (2 mg·mL^−1^) (**a**) and the evolution of the diameters of the nanoparticles of the tBP3 versus temperature (2 mg·mL^−1^) (**b**).

**Scheme 2:**
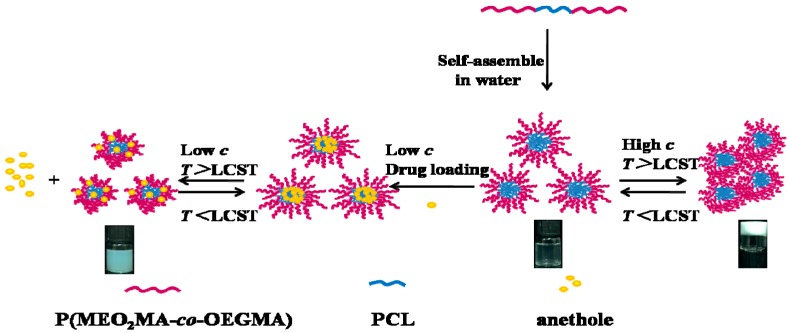
Schematic representation of the self-assembled thermo-sensitive core–shell micelles for temperature-stimulated drug release and gelation of tPBs.

The particle sizes and morphologies of the copolymers in aqueous solution were investigated by DLS and TEM measurements. Figure 4 exhibits the DLS data on the diameters of the micelle prepared from copolymer in aqueous solution (2 mg·mL^−1^). In Figure 4a, all block copolymers formed unimodal size distribution curves, which indicate the formation of relatively similar sized miceller aggregates from the corresponding block copolymers. In addition, the particle average sizes for the tBP1, tBP2, and tBP3 micelles were 155.5, 149.1 and 129.5 nm, respectively, in Figure 4a. It clearly indicates that the hydrodynamic diameter of the micelle formed from P(MEO_2_MA-*co*-OEGMA)-*b*-PCL-*b*-P(MEO_2_MA-*co*-OEGMA) triblock copolymers in water at 25 °C (below LCST) decreases with the increase in the chain length of hydrophilic P(MEO_2_MA-*co*-OEGMA) block. Such observation may be due to when the PCL core remains constant, the higher hydrophilic block length in the polymer, the stronger interactions of the longer hydrophilic groups among themselves at the shell part of the micelles. The observation is in good agreement with the earlier reports [27,29]. The evolution curve of the diameters of the nanoparticles of the tBP3 versus temperature is presented in Figure 4b, which shows that the diameter of polymer micelles increases first, then decreases and ultimately remains stable with the increase of temperature. At room temperature, the copolymer self-assembles into micelles with hydrophobic PCL as the core and hydrophilic segments P(MEO_2_MA-*co*-OEGMA) as the shell, and the particle size was about 130 nm. When the temperature rises to its LCST value, the particle size reaches the maximum of 350 nm, causing the shell to shrink and package around the core, due to the hydrogen bond interaction gradually beginning to weaken. The micelles turned into nanoparticles that existed in a loose state. When the temperature exceeds the LCST of the triblock copolymer, due to the hydrogen bond interaction completely vanishing and the interaction between the hydrophobic group and hydrophobic group enhancing, the water in the loose structures will be squeezed out, then the structures collapse and the particle sizes decrease slightly (as shown in Scheme 2). In addition, the PDI values of tBP3 for the DLS data as a function of the temperature is shown in Table S2.

**Figure 5:**
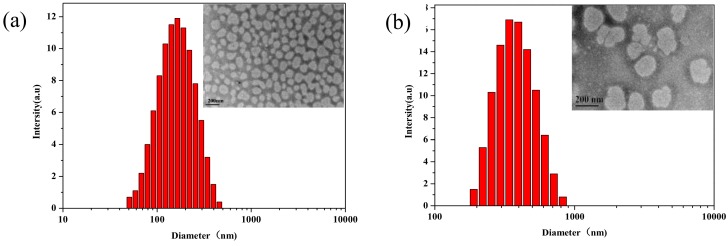
TEM images and size distribution profiles of tBP3 micelles in distilled water, determined by DLS measurement at 25 °C (**a**) and 35 °C (**b**) (2 mg·mL^−1^).

Figure 5 illustrates the size distributions and morphologies of the particles prepared from tBP3 aqueous solution (2 mg·mL^−1^) at 25 and 35 °C. The morphology of tBP3 polymeric micelles was also observed by TEM as shown in Figure 5, which further confirmed that the copolymer could self-assemble to nanosized spherical micelles in water. About 300 nm of the particle size at 35 °C was larger than the 100 nm at 25 °C, which was consistent with the changing trend of the data measured by DLS. Nevertheless, the mean diameter observed by TEM was smaller than that from DLS due to the shrinkage of the P(MEO_2_MA-*co*-OEGMA) segments during the drying for TEM and the relative extension in aqueous solution for DLS.

### 3.5. Thermo-Induced Sol-Gel Transitions of the Triblock Copolymers 

**Figure 6:**
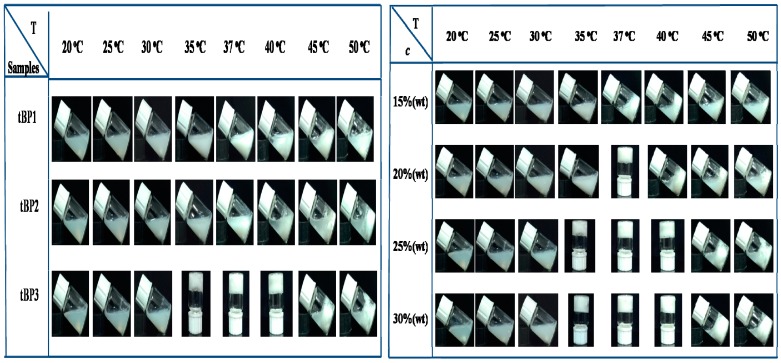
Photographs of P(MEO_2_MA-*co*-OEGMA)-*b*-PCL-*b*-P(MEO_2_MA-*co*-OEGMA) aqueous solutions at different temperature: (**left**) tBP1, tBP2, and tBP3 aqueous solutions (25 wt %) at different temperatures; and (**right**) various concentrations of tBP3 aqueous solutions at different temperatures.

The sol-gel transition of the triblock copolymers aqueous solutions was demonstrated by vial inversion tests induced by gradually changing temperature. Figure 6 (left) showed sol-gel transition photographs for different degrees of polymerization of the triblock copolymers aqueous solutions at a concentration of 25 wt % under various temperatures. All samples were translucent and flowing freely at 20, 25, and 30 °C. The tBP3 aqueous solutions had been converted into a white gel when the temperature rises to 35 °C. However, the tBP1 and tBP2 aqueous solutions did not show a sol-to-gel transition. As the temperature continued to increase to 50 °C, the gel state of tBP3 underwent gel-syneresis transition, white precipitations for the tBP1 and tBP2 solutions were observed instead of forming hydrogels in the process of the rising temperature. The gel formation of the polymer was due to the P(MEO_2_MA-*co*-OEGMA) blocks being incorporated as the associative blocks and forming a three-dimensional network through hydrophobic interactions above the LCST. The result indicates that the sol-gel transition decreases upon increasing the hydrophobic block length and the long hydrophilic blocks could prevent precipitation of the polymeric system. It also shows that tBP1, tBP2, and tBP3 solutions can respond to temperature.

Figure 6 (right) showed photographs of the sol-gel transition for various concentrations of the tBP3 aqueous solution under various temperatures. The observed photographs indicate the concentration of tBP3 more than 20%, which can form a gel induced by temperature from 25 to 50 °C. It also indicated that the gelling temperature *T*_sol-gel_ with different concentrations was decreasing as concentrations increased, and the gels could remain stable in a certain temperature range after the *T*_sol-gel_. The above results show that the sol-gel transition can be achieved under temperature induction at high concentration of the polymer solution, so this hydrogel system may be used as a carrier for hydrophobic drug. Here, similar conclusions are also reported in the literature [25,26].

### 3.6. Drug Loading and In Vitro Release Studies

Amphiphilic polymer is an important drug carrier, because the amphiphilic block copolymers can self-assemble into the core-shell structure micelles in aqueous solution with a hydrophobic core and a hydrophilic shell. The hydrophobic core can load hydrophobic drugs, and the hydrophilic shell can play a protective role resulting in their bioavailability and circulation time in blood being effectively improved [34,35]. In this work, anethole, as a model of the hydrophobic, drug was loaded into the core of P(MEO_2_MA-*co*-OEGMA)-*b*-PCL-*b*-P(MEO_2_MA-*co*-OEGMA) polymeric micelles to study drug loading and release efficiency of the novel copolymer micelles.

The drug loading (DL) and encapsulation efficiency (EE) of micelles were obtained by using UV absorbance. As shown in Table S3 when the initial drug amount increases from 2 to 4 mg, the DL of tBP3 increased from 5.1% to 7.9% and the corresponding EEs were 56.1% and 47.4%, respectively. Table S4 gives the DL and EE of the tBP1, tBP2, and tBP3 micelles whose initial drug amount were 2 mg. The DL decreased from 5.7% to 5.1%, and the corresponding EE decreased from 63.3% to 56.1% from sBP1 to sBP3. The results shown in Tables S3 and S4 indicated that the LC and EE values were higher for micelles formed by polymers with more PCL content. The higher the PCL content in the copolymers, the larger the vacancy volume in the micelles to encapsulate the anethole molecule by hydrophobic interaction. All the results indicated that higher degrees of LC and EE of anethole-loaded micelles could be achieved.

**Figure 7:**
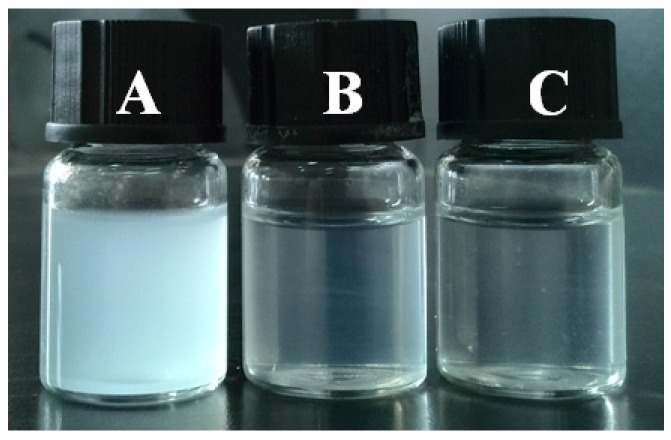
Photographs of anethole solution: (**A**) 0.1% anethole in PBS; (**B**) A_1_-tBP3 (DL = 5.1%); and (**C**) A_2_-tBP3 (DL = 7.9%).

**Figure 8:**
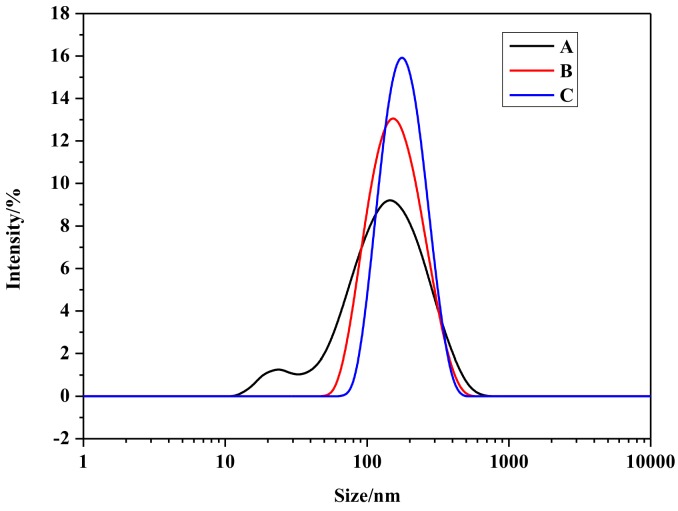
Intensity size distribution: (**A**) tBP3 (DL = 0); (**B**) A_1_-tBP3 (DL = 5.1%) and (**C**) A_2_-tBP3 (DL = 7.9%).

The digital optical pictures of anethole-loaded micelles in PBS at 1 mg mL^−1^ are shown in Figure 7. In PBS, pure anethole forms turbid solution of suspended particles due to the poor solubility (Figure 7A). In contrast, in the case of anethole-loaded micelles (Figure 7B,C), a homogenous and transparent solution is obtained indicating that hydrophobic anethole was successfully loaded inside the polymeric micelles. Moreover, the intensity size distributions of anethole-free micelles and anethole-loaded micelles are shown in Figure 8. Compared with tBPs polymeric micelles, the significantly-increased diameters of anethole-loaded micelles also indicated that anethole was loaded into the micelles successfully (shown in Tables S3 and S4).

**Figure 9:**
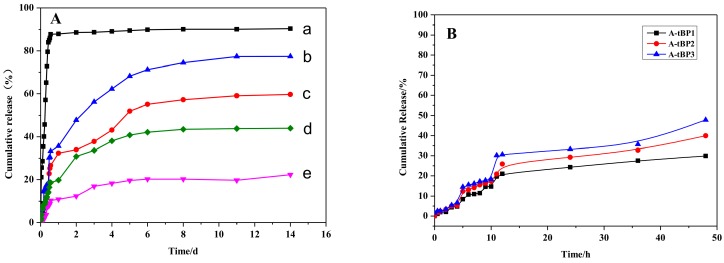
The drug release profiles (**A**) of anethole-loaded micelles: (a) pure anethole; (b) A_1_-tBP3 (DL = 5.1%) at 37 °C; (c) A_1_-tBP3 (DL = 5.1%) at 25 °C; (d) A_2_-tBP3 (DL = 7.9%) at 37 °C; (e) A_2_-tBP3 (DL = 7.9%) at 25 °C; and (**B**) of anethole-loaded micelles of A-tBP1 (DL = 5.7%), A-tBP2 (DL = 5.3%), and A-tBP3 (DL = 5.1%) at 37 °C.

The drug release was performed under physiological conditions (PBS, pH 7.4) at 25 and 37 °C, respectively. As shown in Figure S3, a linear standard curve was gained between the UV absorbance and anethole concentration with a correlation coefficient of 0.9998. The in vitro release profiles of free anethole and anethole-loaded tBP3 polymeric micelles (with initial drug amounts of 2 and 4 mg, respectively) are shown in Figure 9A. As can be shown, pure anethole was released up to 85% in 10 h, while the anethole–loaded micelles showed much slower and sustained release. Drug release is greatly influenced by temperature. The cumulative release of anethole from A_1_-tBP3 (DL = 5.1%) and A_2_-tBP3 (DL = 7.9%) reaches 35.27% and 19.75% after one day at 37 °C, in contrast to 32.34% and 10.90% at 25 °C. In addition, the release at 8 day reaches 74.52% and 57.21% at 37 and 25 °C for A_1_-tBP3, and 42.46% and 20.28% for A_2_-tBP3. After 14 days, the cumulative release of anethole was 77.42% and 43.99% at 37 °C for A_1_-tBP3 and A_2_-tBP3, respectively. From the above data we can draw a conclusion that drug release is favored at 37 °C. At 25 °C (*T* < LCST) the highly-hydrated P(MEO_2_MA-*co*-PEGMA) segments stabilize the hydrophobic-hydrophilic core–shell structure of micelles, allowing a small amount of drug to diffuse out from the micelles. At 37 °C (*T* > LCST), the P(MEO_2_MA-*co*-PEGMA) shell become hydrophobic, and the micellar core-shell structure was deformed, leading to the hydrophobic drug incorporated in core diffused out quickly from the micelles (Scheme 2). As shown in Figure 9B, the cumulative release of anethole from A-tBP1 (DL = 5.7%), A-tBP2 (DL = 5.3%) and A-tBP3 (DL = 5.1%) reaches 29.88%, 39.93%, and 47.80% after 48 h at 37 °C, respectively. The results (Figure 9A,B) indicated that high drug loading exhibits slower drug release, possible because the solubility of anethole in the release medium is a limiting factor. Additionally, the drug-loaded micelles with high drug loading had a more compact core structure due to the stronger interaction between the drug and hydrophobic chains, which could disfavor drug diffusion. All in all, in release profiles the anethole-loaded micelles underwent a relatively fast anethole release, then reached their slow release stages, indicating that the anethole-loaded polymeric micelles have favorable stability during systematic circulation under physiological conditions.

## 4. Conclusions

Well-defined linear amphiphilic temperature-responsive triblock copolymers P(MEO_2_MA-*co*-OEGMA)-*b*-PCL-*b*-P(MEO_2_MA-*co*-OEGMA) (tBPs) were synthesized by ROP and RAFT. The LCST of the synthesized copolymer increases in aqueous solution with increasing the content of OEGMA. The LCST of the synthesized copolymer could be adjusted close to human physiological temperature at 35 (±1) °C via controlling the feed ratio of the MEO_2_MA and OEGMA comonomers. The critical micelle concentration (CMC) value of the micelle increased with the increase in the chain length of the P(MEO_2_MA-*co*-OEGMA) block. In addition, the self-assembly of triblock copolymer micelles in aqueous solutions showed low CMC under physiological conditions (pH = 7.4), which is beneficial to drug delivery due to the stability of micelles in long circulation in the bloodstream upon dilution under physiological conditions. DLS results show a strong increase of anethole-polymer micellar size, suggesting anethole was successfully loaded inside polymeric micelles and the strong interaction between the drug and copolymers with the hydrophobic block. In vitro drug release revealed that micelles with lower drug loading show faster release than micelles with high drug loading. Hence, the thermo-responsive triblock copolymers can also be used as a carrier of hydrophobic drugs and have excellent biocompatibility and a well-controlled release performance.

## Supplementary Materials

The following are available online at http://www.mdpi.com/2073-4360/10/2/214/s1. Figure S1: FT-IR spectra of CTA, Figure S2: 1H NMR spectra (A) of CTA (a), OH-*b*-PCL-*b*-OH (b) and CTA-*b*-PCL-*b*-CTA (c); (B) of the triblock copolymer of P(MEO2MA-*co*-OEGMA)-*b*-PCL-*b*-P(MEO2MA-*co*-OEGMA) in CDCl3 (a) and D2O (b) at room temperature, Figure S3: Calibration curve for anethole—loaded drug release, Table S1: Characterization of various copolymers, Table S2: The PDI values of tBP3 for the DLS data as a function of the temperature, Table S3: Properties of tBP3 micelles with and without anethole, Table S4: The properties of tBPs micelles with and without anethole.

## Abbreviations

AIBN	azobisisobutyronitrile
CTA	*S*-1-dodecyl-*S*′-(α,α′-dimethyl-α″-acetic acid) trithiocarbonate chain transfer agent
CMC	critical micellization concentration
CGT	gel-to-sol transition
DLS	dynamic light scattering
DMAP	4-(*N*,*N*-dimethylamino)pyridine
DP	degree of polymerization
EDC	*N*-(3-(dimethylamino) propyl)-*N*-ethylcarbodiimide hydrochloride
FDA	US Food and Drug Administration
LCST	lower critical solution temperature
MEO_2_MA	2-(2-methoxy ethoxy) ethylmethacrylate
OEGMA	oligo (ethylene glycol) methacrylate
PBS	phosphate-buffered solution
PCL	poly(*ε*-caprolactone)
PEG	poly(ethyleneglycol)
PNIPAAm	poly(*N*,*N*-dimethylacrylamide)
ROP	ring-opening polymerization
RAFT	reversible addition-fragmentation chain-transfer polymerization
Sn(Oct)_2_	stannous octoate
