# Supplementary material for: Novel Amphiphilic, Biodegradable, Biocompatible, Thermo-Responsive ABA Triblock Copolymers Based on PCL and PEG Analogues via a Combination of ROP and RAFT: Synthesis, Characterization, and Sustained Drug Release from Self-Assembled Micelles
Source: Polymers (Basel). 2018 Feb 22;10(2):214. doi: 10.3390/polym10020214 (PMC6415266; doi:10.3390/polym10020214)
Supplement: Supplementary file 1 [file polymers-10-00214-s001.pdf]

Supplementary Materials

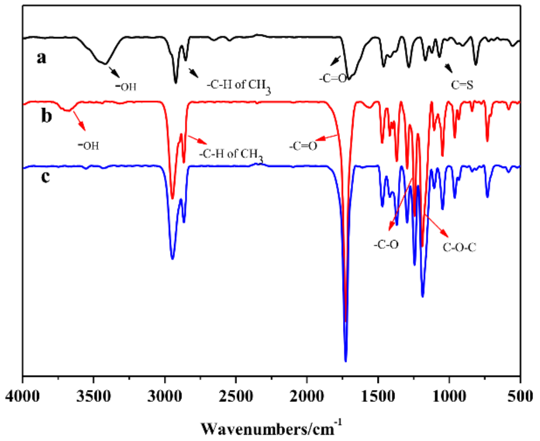

Figure S1. FT-IR spectra of CTA (**a**), OH-*b*-PCL-*b*-OH (**b**), and CTA-*b*-PCL-*b*-CTA (**c**).

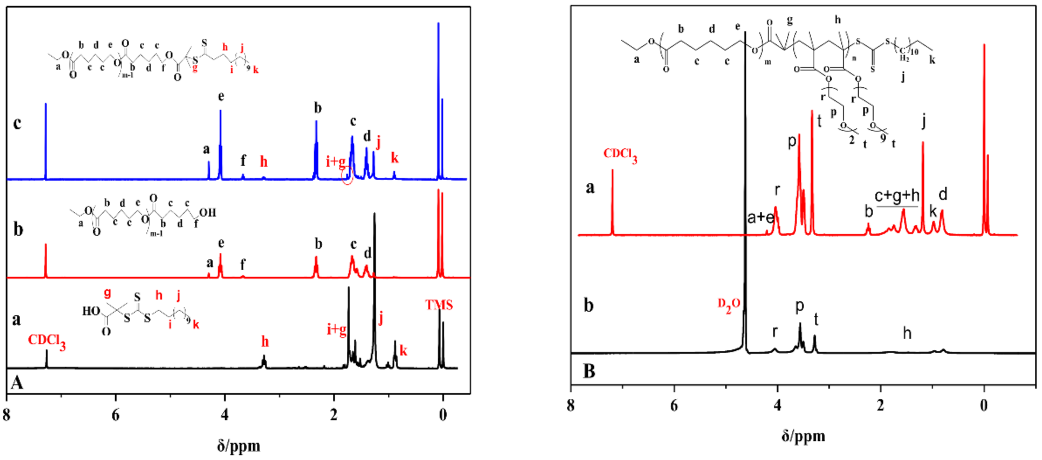

Figure S2.^{1}H NMR spectra :(**A**) of CTA (**a**), OH-*b*-PCL-*b*-OH (**b**) and CTA-*b*-PCL-*b*-CTA (**c**); (**B**) of the triblock copolymer of P(MEO2MA-*co*-OEGMA)-*b*-PCL-*b*-P(MEO2MA-*co*-OEGMA) in $CDCl_3$ (**a**) and D_2O (**b**) at room temperature.

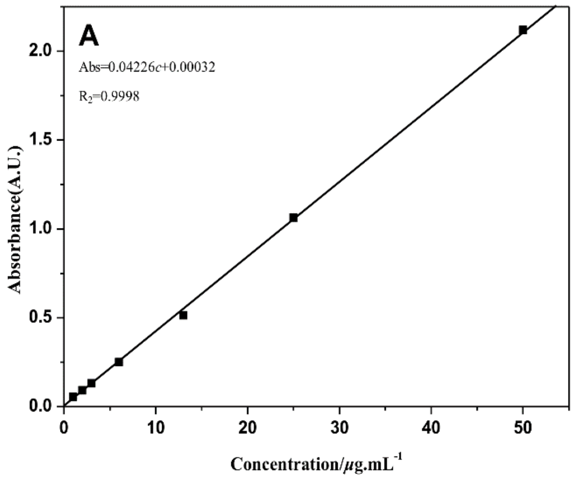

Figure S3. Calibration curve for anethole—loaded drug release.

Table S1. Characterization of various copolymers.

Samples	DPCL [a]	$[M]_0$:$[O]_0$ [b]	DP [c]	M_n, Theory [d] (g/mol)	M_n, GPC [e] (g/mol)	PDI [e]
tB1	30	-	-	3486	3716	1.54
tB2	30	-	-	4178	4349	1.46
tBP1	30	97:3	220	47,479	54,775	1.16
tBP2	30	97:3	250	53,384	57,485	1.06
tBP3	30	97:3	280	59,289	61,571	1.01
tBP4	30	92:8	250	56,969	59,605	1.04
tBP5	30	87:13	250	60,553	64,450	1.11

tB1 and tB2 represent the OH-PCL-OH and CTA-PCL-CTA. tBP represents theP(MEO_2MA-*co*-OEGMA)-*b*-PCL-*b*-P(MEO_2MA-*co*-OEGMA).DPCL [a]is the DP of PCL segments. $[M]_0$:$[O]_0$[b] is the initial molar ratio of MEO_2MA and OEGMA,DP [c]is the DP of monomers' segments. [d] Calculated theory analysis from the feed ratio of monomers to macro-chain transfer agents. [e] Determined by GPC with THF as the eluent and polystyrene as the standard.

Table S2. The PDI values of tBP3 for the DLS data as a function of the temperature.

T (°C)	25	27	29	31	33	35
	37	39	41	43	45	50
Size-average (*d*/nm) [a]	129.5	130.6	132.6	253.8	341.2	348.6
	343.9	337.4	328.8	310.1	310.7	297.4
PDI [a]	0.247	0.254	0.225	0.081	0.027	0.031
	0.128	0.017	0.052	0.115	0.124	0.250

[a] Determined by DLS in water(2mg mL^{-1}).

Table S3. Properties of tBP3 micelles with and without anethole.

Samples	Drug/Polymer [a] (mg/mg)	Size-Average [b] (*d*/nm)	PDI [b]	DL [c] (wt%)	EE [d] (wt%)
tBP3	0/20	105.8	0.300	-	-
A_1-tBP3	2/20	144.1	0.157	5.1	56.1
A_2-tBP3	4/20	162.5	0.150	7.9	47.4

[a] In feed; [b] Determined by DLS in water(1mg mL^{-1}); [c] Determined by UV absorbance.

TableS4. The properties of tBPs micelles with and without anethole.

Samples	Blank Size-Average [a] (*d*/nm)	PDI [b]	A-Loaded Micelles Size-Average [a] (*d*/nm)	PDI [b]	DL [c] (wt%)	EE [d] (wt%)
tBP1	149.9	0.291	206.9	0.171	5.7	63.3
tBP2	119.9	0.320	163.9	0.194	5.3	58.9
tBP3	105.8	0.300	144.1	0.157	5.1	56.1

[a,b] Determined by DLS in water in water(1mg mL^{-1}); [c] Determined by UV absorbance.
